# Acquired neuromyotonia in thymoma‐associated myasthenia gravis: a clinical and serological study

**DOI:** 10.1111/ene.13922

**Published:** 2019-03-25

**Authors:** M. Gastaldi, A. De Rosa, M. Maestri, E. Zardini, S. Scaranzin, M. Guida, P. Borrelli, O. E. Ferraro, V. Lampasona, R. Furlan, S. R. Irani, P. Waters, B. Lang, A. Vincent, E. Marchioni, R. Ricciardi, D. Franciotta

**Affiliations:** ^1^ Neuroimmunology Laboratory IRCCS Mondino Foundation Pavia Italy; ^2^ Neurology Unit Department of Clinical and Experimental Medicine University of Pisa Pisa Italy; ^3^ Unit of Biostatistics and Clinical Epidemiology University of Pavia Pavia Italy; ^4^ Division of Genetics and Cell Biology Genomic Unit for the Diagnosis of Human Pathologies San Raffaele Scientific Institute Milan Italy; ^5^ Division of Neuroscience INSPE San Raffaele Scientific Institute Milan Italy; ^6^ Oxford Autoimmune Neurology Group Nuffield Department of Clinical Neurosciences University of Oxford Oxford UK; ^7^ Nuffield Department of Clinical Neurosciences University of Oxford Oxford UK; ^8^ Neuroncology and Neuroinflammation Unit IRCCS Mondino Foundation Pavia Italy

**Keywords:** autoantibodies, DCC antibodies, myasthenia gravis, neuromyotonia, paraneoplastic neurological syndrome, thymoma, UNC5A antibodies

## Abstract

**Background and purpose:**

Acquired neuromyotonia can occur in patients with thymoma, alone or in association with myasthenia gravis (MG), but the clinical prognostic significance of such comorbidity is largely unknown. The clinico‐pathological features were investigated along with the occurrence of neuromyotonia as predictors of tumour recurrence in patients with thymoma‐associated myasthenia.

**Methods:**

A total number of 268 patients with thymomatous MG were studied retrospectively. Patients with symptoms of spontaneous muscle overactivity were selected for autoantibody testing using immunohistology for neuronal cell‐surface proteins and cell‐based assays for contactin‐associated protein 2 (CASPR2), leucine‐rich glioma inactivated 1 (LGI1), glycine receptor and Netrin‐1 receptor antibodies. Neuromyotonia was diagnosed according to the presence of typical electromyography abnormalities and/or autoantibodies against LGI1/CASPR2.

**Results:**

Overall, 33/268 (12%) MG patients had a thymoma recurrence. Five/268 (2%) had neuromyotonia, four with typical autoantibodies, including LGI1 (*n* = 1), CASPR2 (*n* = 1) or both (*n* = 2). Three patients had Netrin‐1 receptor antibodies, two with neuromyotonia and concomitant CASPR2+LGI1 antibodies and one with spontaneous muscle overactivity without electromyography evidence of neuromyotonia. Thymoma recurrence was more frequent in those with (4/5, 80%) than in those without (28/263, 10%, *P* < 0.001) neuromyotonia. Neuromyotonia preceded the recurrence in 4/5 patients. In univariate analysis, predictors of thymoma recurrence were age at thymectomy [odds ratio (OR) 0.95, 95% confidence interval (CI) 0.93–0.97], Masaoka stage ≥IIb (OR 10.73, 95% CI 2.38–48.36) and neuromyotonia (OR 41.78, 95% CI 4.71–370.58).

**Conclusions:**

*De novo* occurrence of neuromyotonia in MG patients with previous thymomas is a rare event and may herald tumour recurrence. Neuronal autoantibodies can be helpful to assess the diagnosis. These observations provide pragmatic risk stratification for tumour vigilance in patients with thymomatous MG.

## Introduction

Acquired neuromyotonia (NMT) is characterized by spontaneous and continuous muscle overactivity resulting from peripheral nerve hyperexcitability 1, 2. It is associated with antibodies to contactin‐associated protein 2 (CASPR2), leucine‐rich glioma inactivated 1 (LGI1) or both in around 40% of cases and shows good response to plasmapheresis 3, 4. These antibodies target neuronal cell‐surface epitopes, and thus they are probably pathogenic 5. NMT can be associated with myasthenia gravis (MG) with unknown frequency 6. In these patients, peripheral nerve hyperexcitability symptoms, such as fasciculations and cramps, might be overlooked because of the coexisting defect of neuromuscular transmission, or may be misinterpreted as side effects of anti‐acetylcholinesterase drugs 7. Neurophysiological assessments and testing for NMT‐specific autoantibodies can aid the diagnosis.

Thymoma, an epithelial tumour typically associated with paraneoplastic neurological syndromes 8, occurs in 10%–15% of patients with MG in western countries 9 and in up to 20% of those with NMT 1. Its recurrence, which affects around 15% of thymectomized patients 10, 11, is insidious and has a poor prognosis, making early recognition a fundamental goal.

The association between MG, NMT and thymoma is a very rare condition. The onset of NMT that heralded thymoma recurrence has been described in a single patient with MG 12. Recently, Torres‐Vega *et al*. identified new antibodies to neuronal cell‐surface antigens, the Netrin‐1 receptors deleted in colon cancer (DCC) and uncoordinated‐5A (UNC5A), in patients with MG, NMT or both 13.

To assess the relationships between NMT, MG and thymoma, a large series of thymoma‐associated MG patients were studied. Serum autoantibodies to neuronal cell‐surface antigens were determined and predictors of thymoma recurrence were sought.

## Methods

### Subjects and materials

Overall, 268 consecutive acetylcholine receptor antibody seropositive MG patients with thymoma referred to the outpatient clinics of Pisa (Italy) from 1990 to 2016 were included. Demographic and clinico‐pathological information was collected retrospectively. MG grading was assessed before thymectomy and at the end of follow‐up according to the MG Foundation of America classification 14. Thymoma staging was performed according to the World Health Organization 15 and Masaoka system 16. Thymoma recurrence was defined as the local, regional or distant reappearance of the tumour after complete eradication 17. Sera from 23 patients showing symptoms and signs of spontaneous muscle overactivity involving at least two skeletal districts, collected and stored at −20°C during follow‐up, were tested. Patients were defined as having NMT if the following criteria were fulfilled: (i) the presence of cramps and/or muscle twitching (myokymia and/or fasciculations) affecting at least two skeletal regions; (ii) no pyridostigmine treatment, or symptom persistence after the drug suspension 18; (iii) electromyography (EMG) compatible with NMT (myokymic/neuromyotonic discharges) 4 or, in patients without EMG available, seropositivity for CASPR2 and/or LGI1 antibodies, which can typically be associated with NMT 19.

The study was approved by the Institutional Review Boards of the Institutes of Pisa and Pavia. Informed consent for antibody studies was obtained from all the patients.

### Antibody testing

Immunohistochemistry on rat whole brain, with a protocol of tissue light fixation that preserves the native antigen conformation, was the screening technique for neuronal cell‐surface antibody detection 20. Human embryonic kidney (HEK293T) cells were used for live cell‐based assays (CBAs) for antibodies to CASPR2, LGI1, glycine receptor and UNC5A (materials and procedures are described in Data S1). A fixed CBA was used for DCC antibodies 21. Sera were also tested for onconeural antibodies on primate cerebellum (indirect immunofluorescence kit, Euroimmun, Lubeck, Germany) and for voltage‐gated potassium channel (VGKC) complex antibodies with a radioimmunoassay at the Oxford Neuroimmunology Laboratory (John Radcliffe Hospital, Oxford, UK) 22. To validate the newly established UNC5A and DCC CBA, 152 control sera were used from patients with thymoma without neurological symptoms (*n* = 6), MG without thymoma (*n* = 28), other neurological diseases (*n* = 118; Alzheimer disease, 74; normal pressure hydrocephalus, 44) and from 17 healthy individuals. Results were visualized on a fluorescence or light microscope and considered positive if the independent judgements of two blinded‐to‐clinical‐information investigators (M.G., E.Z.) were concordant. For positive samples, end‐point titrations were obtained using serial dilutions.

### Statistical analysis

Qualitative variables were summarized as percentages and quantitative variables as median with interquartile ranges (IQRs). Differences in quantitative variables were tested using the *t* test or the analogous nonparametric method (Mann‐Whitney test), and for qualitative variables the chi‐squared or Fisher's exact test. To evaluate associations, univariate odds ratios (ORs) with 95% confidence interval (CI) were calculated. *P* values ≤0.05 were considered significant (two‐sided). All analyses were performed with STATA/SE for Windows, v14 (www.stata.com).

## Results

### Patients with thymomatous MG

Table 1 shows the clinico‐demographic characteristics of the 268 patients. The median age at thymectomy was 49 years (IQR 39–61) and the male:female ratio was 1:1; 94% of patients had generalized MG and 12% severe muscular weakness in bulbar, spinal or both districts. MG preceded the detection of a thymoma in 93% of the cases; 95% of the patients had undergone thymectomy by the trans‐sternal approach and 5% by the minimally invasive approach; the most frequent thymoma histology, B2 15, was found in 27% of the cases. No patient had a thymic carcinoma. 152/268 patients (57%) received additional chemotherapy and/or radiotherapy. According to surgical reports, all patients underwent a radical tumour excision.

**Table 1:** Clinico‐demographic data of thymomatous myasthenia gravis patients

Patients, *n* (%)	All patients, 268 (100)	No thymoma recurrence, 236 (89)	Thymoma recurrence, 33 (12)	*P* value	Univariate, OR (CI)	*P* value
Months of follow‐up, median (IQR)	41.5 (8–88)	33 (7–77)	87 (58–146)	<0.001	–	–
Age at thymectomy, years, median (IQR)	49 (39–61)	51 (41–62)	40 (31–47)	<0.001	0.95 (0.93–0.97)	<0.001
Males, *n* (%)	134 (50)	121 (52)	13 (39)	0.193	0.61 (0.29–1.29)	0.194
Thymoma histology (WHO; ≥B2), *n* (%)	171/264* (65)	145/231* (63)	26 (79)	0.072	2.20 (0.91–5.33)	0.072
Thymoma grading(Masaoka, ≥IIb), *n* (%)	156/252* (62)	127/221* (58)	29/31 (94)	<0.001	10.73 (2.38–48.36)	<0.001
Age at MG onset, median (IQR)	49 (38–61)	50 (40–62)	38 (29–45)	<0.001	0.94 (0.92–0.96)	<0.001
MG grading before thymectomy (MGFA ≥III), *n* (%)	84 (31)	73 (31)	11 (31)	0.792	1.11 (0.51–2.41)	0.793
MG onset before thymectomy, *n* (%)	248 (93)	220 (94)	28 (85)	0.073	0.38 (0.13–1.14)	0.073
MG duration (months) before thymectomy, median (IQR)	5 (2–13)	5 (2–12)	5 (2–26)	0.487	1.01 (1.00–1.01)	<0.001
NMT, *n* (%)	5 (2)	1 (0.4)	4 (12)	<0.001	32.28 (3.15–330.05)	<0.001

CI, confidence interval; IQR, interquartile range; MG, myasthenia gravis; MGFA, MG Foundation of America classification; NMT, neuromyotonia; OR, odds ratio; WHO, World Health Organization score; *Number of available patients.

### Patients with neuromyotonia and serological study

The characteristics of the 23 patients with thymomatous MG and spontaneous muscle overactivity are shown in Table S1. Twelve of them underwent EMG. Due to this shortcoming, only five patients fulfilled our criteria for NMT. Table 2 shows their demographic and clinical paraclinical features. EMG evidence of myokymic discharges was found in two patients, one without LGI1/CASPR2 autoantibodies. NMT symptoms included myokymia (*n* = 2), fasciculations (*n* = 2) or both (*n* = 1) and involved more commonly the lower and/or upper limbs (*n* = 5). Three patients had periocular muscle myokymia and one trunk myokymia. Four patients had accessory sensory symptoms, two reporting burning pain in the lower limbs. A concomitant central nervous system (CNS) involvement was seen in three patients, one with new onset focal seizures and two with a combination of encephalopathy and sleep disorder with or without dysautonomia, who were eventually diagnosed as Morvan syndrome. Four of them had a thymoma recurrence.

**Table 2:** Characteristics of thymomatous myasthenia gravis patients with neuromyotonia

*N*	Sex	Age	MG stage	Thymoma (WHO)	Additional treatment	Timing of recurrence after thymectomy	NMT timing	Main NMT symptoms	Accessory symptoms	CNS symptoms	Antibodies (end‐point titre [1:])	NMT treatment	Response to treatment	NMT electrodiagnosis	Follow‐up time (months)	Final diagnosis	MG outcome
1	F	47	IIA	B2	Chemo/RT	3 and 7 years	6 months before second relapse	Orbicular and abdominal myokymia	Hyperhidrosis	New onset focal seizures	CASPR2 (200)	LEV	Good	EMG not performed	118	NMT	Minimal manifestation
2	F	33	IVB	B2	No	No recurrence	1 month after thymectomy	Ocular myokymia, fasciculations in upper limbs	Paresthesias in lower limbs, hyperhidrosis, arrythmia	None	LGI1 (160)	PNT	Good	No (EMG performed after symptom resolution)	22	NMT	Improved
3	F	38	IIIB	B3	Chemo/RT	3 years	1 year before recurrence	Severe ocular myokymia; diffuse cramps and fasciculations	Paresthesias in lower limbs	None	None	CBZ	Good	Diffuse fibrillation and myokimic discharges (doublets and triplets) in four limbs	83	NMT	Pharmacological remission
4	M	36	IIA	B1	Chemo	3 years	3 months before recurrence	Fasciculations and cramps in lower limbs	Burning pain in lower limbs, hyperhidrosis	Sleep disorder, personality change	CASPR2 (800)+LGI1 (640)+DCC (900)+UNC5A (2700)	PNT, PRG, CBZ	Good	No (EMG performed after symptom resolution)	98	NMT+CNS symptoms (Morvan syndrome)	Pharmacological remission
5	M	57	I	B2	Chemo	9 years	After recurrence	Cramps and fasciculation in lower limbs	Paresthesias and pain in lower limbs	Seizures, sleep disorder, memory impairment	CASPR2 (200)+LGI1 (320)+DCC (300)	None	NA	Diffuse fasciculations and myokimic discharges in lower limbs	165	NMT+CNS symptoms (Morvan syndrome)	Death (from neoplastic disease)

CBZ, carbamazepine; CNS, central nervous system; DCC, deleted in colon cancer; EMG, electromyography; LEV, levetiracetam; MG, myasthenia gravis; Chemo, chemotherapy; NA, not applicable; NMT, neuromyotonia; PNT, phenoytoin; PRG, pregabalin; RT, radiotherapy; UNC5A, uncoordinated‐5A; WHO, World Health Organization score.

**Figure 1:**
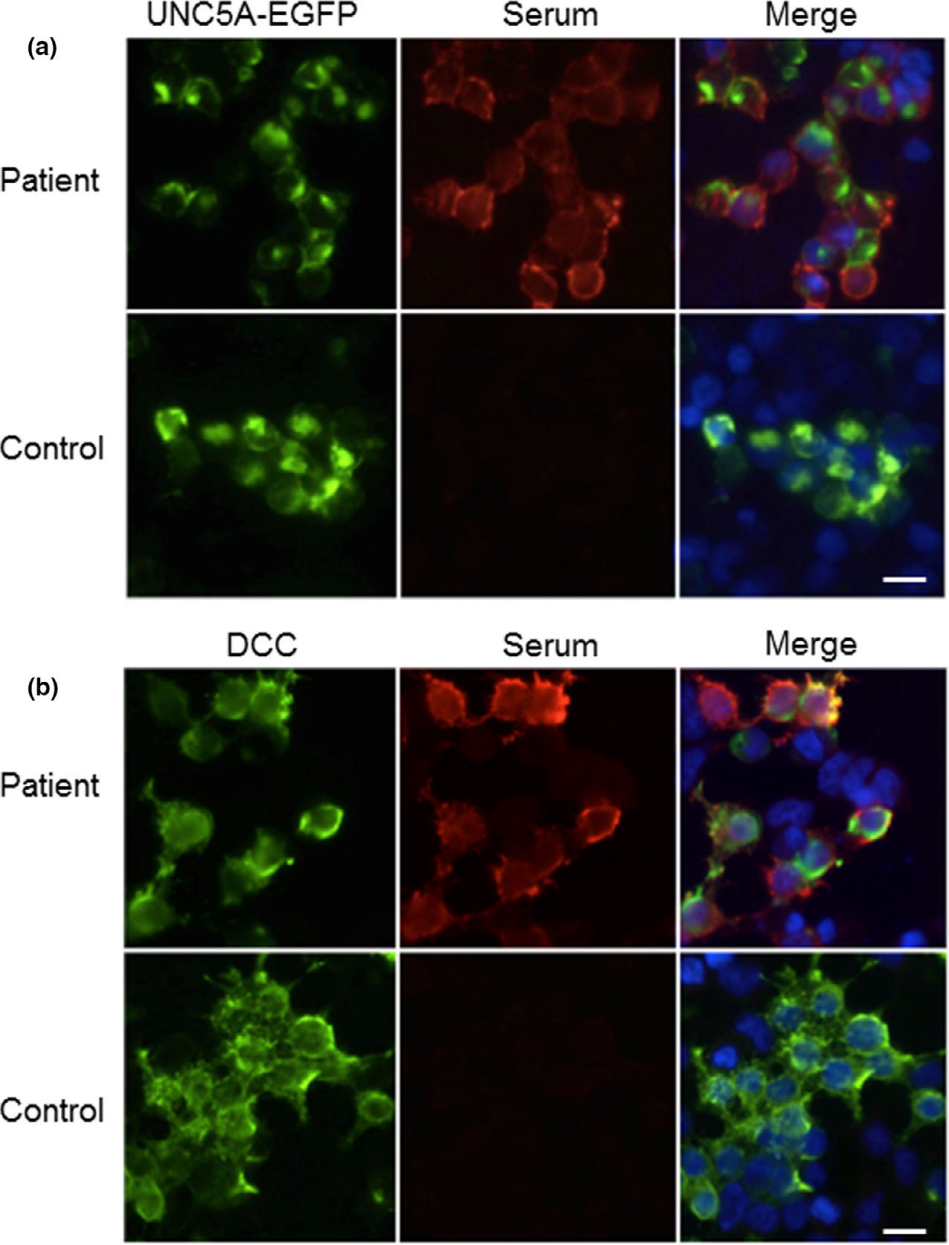
Cell‐based assays for Netrin‐1 receptor antibody detection. HEK293T cells transfected with either enhanced green fluorescent protein (EGFP) tagged UNC5A (a) or commercial fluorescent antibody against DCC (b) (left panels). Immunoglobulin G from patients, but not from controls, bind the cells (central panels), co‐localizing with the EGFP tagged UNC5A or with the DCC antibody (merging, right panels; 4′,6‐diamidino‐2‐phenylindole (DAPI) stained nuclei). Scale bar 20 μm. [Color figure can be viewed at wileyonlinelibrary.com].

As a whole, 6/23 patients had autoantibodies against neuronal cell‐surface proteins. No other autoantibody reactivities to unknown antigens were detected on immunohistochemistry screening (Data S1). CASPR2 and/or LGI1 antibodies were found in four patients diagnosed as NMT. Amongst these, two patients showed polyreactivities that included the two Netrin‐1 receptor antibodies (CASPR2+LGI1+DCC+UNC5A and CASPR2+LGI1+DCC; Fig. 1), both diagnosed as Morvan syndrome. One patient with periocular myokymia and fasciculations in the four limbs had isolated antibodies to the Netrin‐1 receptor DCC. EMG was not performed in the acute phase, so the patient could not be diagnosed as NMT. Finally, one patient with limbic encephalitis, fasciculations in the lower limbs and an EMG compatible with cramps fasciculation syndrome was positive for α‐amino‐3‐hydroxy‐5‐methyl‐4‐isoxazolepropionic acid receptor (AMPAR) antibodies. His thymoma recurred concomitantly with the onset of both the spontaneous muscle overactivity and limbic encephalitis.

All control sera were negative for DCC and UNC5A antibodies (additional information on antibody results is given in Supporting information, Figure S1). All the LGI1/CASPR2 antibody seronegative patients were negative for VGKC antibodies. All the samples were negative for onconeural antibodies and for glycine receptor antibodies.

### Predictors of thymoma recurrence

During follow‐up, 33/268 (12%) patients developed a recurrence of thymoma after a median time of 46.1 months (IQR 26.5–63.5), and 18/33 had more than one recurrence. Table 1 reports the clinico‐pathological factors associated with tumour recurrence. In univariate analysis, younger age at thymectomy (OR 0.95, 95% CI 0.93–0.97), higher staging score of the original thymoma (Masaoka stage ≥IIb, OR 10.73, 95% CI 2.38–48.36) and coexistence of NMT (OR 41.78, 95% CI 4.71–370.58) predicted tumour recurrence. No difference in the frequency of tumour recurrence was found when comparing thoracotomy and minimally invasive surgery. Notably, the onset of spontaneous muscle overactivity preceded the discovery of thymoma recurrence in 4/5 NMT patients and was concomitant with the limbic encephalitis in the AMPAR‐antibody‐positive patient. The latency between neurological symptom onset and thymoma recurrence ranged between 0 and 12 months. The only NMT patient without tumour recurrence had a follow‐up shorter than that of the other four patients (22 vs. 119.5 months, median value; range 83–165 months). Overall, amongst the 23 patients tested, anti‐neuronal cell‐surface antibodies were detected more frequently in those with recurrent (4/6, 67%) than in those with monophasic thymoma (4/17, 24%), but this difference did not reach statistical significance (*P* = 0.13).

## Discussion

This study focused on the clinico‐pathological and neuronal autoantibody characterization of patients with MG, NMT and thymoma, extrapolated from a very large series of patients with MG and thymoma who, in turn, were investigated for tumour‐related prognostic factors. Younger age at thymectomy and the occurrence of NMT were identified as novel predictors of thymoma recurrence. In addition, it was confirmed that the recently identified autoantibodies to Netrin‐1 receptors can be found in thymoma‐associated MG with NMT/Morvan syndrome comorbidity, mainly as co‐reactivities.

Due to the retrospective nature of the study, a suggestive EMG was not a mandatory requirement for NMT diagnosis, which could alternatively be assessed if typical autoantibodies (CASPR2 and/or LGI1) were detected. This occurred in three patients: one patient did not undergo EMG, whilst in two patients EMG was normal, probably because the examination was performed after the introduction of sodium‐channel‐blocking drugs that completely resolved the muscle overactivity symptoms. In fact, all three patients had clinical features highly suggestive of NMT involving at least two skeletal districts and, in one of them, the presence of CNS symptoms led to the diagnosis of Morvan syndrome. Previous studies compared patients with peripheral nerve hyperexcitability with or without typical EMG features of NMT, concluding that the frequency of VGKC antibodies (including CASPR2 and LGI1 antibodies) is increased in both groups, thus probably implicated in the pathogenesis, and that the EMG features reflect quantitative rather than qualitative differences 18. For these reasons it was decided that, in the presence of clear and subacutely occurring symptoms of spontaneous muscle overactivity, the detection of disease‐typical autoantibodies could sustain the diagnosis of NMT. Such autoantibodies are indeed very specific 23, and their occurrence in patients with thymoma but without features of NMT or of other neurological syndromes is very rare 24. As a further limitation, EMG was not performed in 11/23 patients with spontaneous muscle overactivity symptoms, possibly preventing the diagnosis of NMT in some of them.

In the literature, there is only one report of a patient with overlapping MG, NMT and thymoma that links the co‐occurrence of NMT with the tumour recurrence 12. Our data give statistical weight to this observation and strongly underpin the importance of proper tumour vigilance and screening in patients with MG receiving the diagnosis of NMT and *vice versa*. In analogy with that report 12 and our cases, an additional thymomatous MG patient had a tumour recurrence concomitantly with the occurrence of diffuse fasciculations and limbic encephalitis, with the detection of AMPAR antibodies. Limbic encephalitis has been frequently associated with thymoma, both isolated and in association with MG, but its role as a predictor of thymoma recurrence warrants future studies 25, 26, 27.

In the most common association with MG, but without NMT, thymoma prognosis has been variably reported as better or worse 28, 29. In our study it was found that an advanced Masaoka staging score was a strong predictor of tumour recurrence, in line with the literature data 11, whereas there was no significant association between thymoma histology and tumour recurrence. This apparent discrepancy is probably related to the high representation, in our patients, of the most commonly MG‐associated B2/B3 stage 28. The evaluation of incomplete tumour resection as another predictor of thymoma recurrence was unavailable due to the lack of data on residual thymic tissues.

The subgroup of patients with thymoma‐associated MG and NMT showed reactivities to known (CASPR2/LGI1) and to the recently identified (Netrin‐1 receptors) neuronal cell‐surface antibodies. Cumulatively, they were present in 4/5 patients, a ratio very similar to that found by Torres‐Vega *et al*. (7/9) 13. In our series, the autoantibodies tended to associate with recurrent thymoma. Only two studies reported the presence of Netrin‐1 receptor autoantibodies, more frequently against DCC than UNC5A, and almost invariably coexisting with CASPR2 antibodies in patients with thymoma‐associated Morvan syndrome 21 or comorbid for thymoma‐associated MG and NMT 13. In partial contrast, Netrin‐1 receptor antibodies (DCC plus UNC5A, or DCC alone) coexisted with both CASPR2 and LGI1 antibodies in two patients with Morvan syndrome and thymoma recurrence. Taking into account the Netrin‐1 receptor involvement in CNS synaptic plasticity 30 and in axonal growth and guidance at the paranodes of Schwann cell axons 31, a possible pathogenic role for these autoantibodies is possible, but is purely speculative at present.

Netrin‐1 receptor antibodies seem to be highly specific for paraneoplastic NMT or Morvan syndrome as they were absent in a large series of controls, confirming previously reported data 13. Only one patient with NMT symptoms but without EMG myokymic discharges had isolated DCC, without manifesting thymoma recurrence.

In conclusion, our study suggests that the occurrence of NMT in thymomatous MG patients is a rare event, but can predict insidious thymoma recurrence, providing pragmatic risk stratification for tumour vigilance. Testing for neuronal autoantibodies can be helpful to assess the diagnosis.

## Disclosure of conflicts of interest

AV, SRI and PW are co‐applicants and receive royalties on patent application WO/2010/046716 (UK patent PCT/GB2009/051441) entitled ‘Neurological Autoimmune Disorders’. The patent has been licensed to Euroimmun AG for the development of assays for LGI1 and other VGKC complex antibodies. All other authors have no competing interests to declare.

## Supporting information


**Table S1** Characteristics of thymomatous MG patients with spontaneous muscle overactivity tested for neuronal autoantibodies.
**Figure S1** Staining patterns for LGI1, CASPR2 and AMPAR antibodies on rat brain tissue.
**Data S1** Supplementary methods
