# Supplementary material for: Acquired neuromyotonia in thymoma‐associated myasthenia gravis: a clinical and serological study
Source: Eur J Neurol. 2019 Mar 25;26(7):992–9. doi: 10.1111/ene.13922 (PMC6593867; doi:10.1111/ene.13922)
Supplement: Supplementary file 1 — Table S1 Characteristics of thymomatous MG patients with spontaneous muscle overactivity tested for neuronal autoantibodies. Figure S1 Staining patterns for LGI1, CASPR2 and AMPAR antibodies on rat brain tissue. Data S1 Supplementary methods [file ENE-26-992-s001.docx]

**Supplementary material**

Supplementary methods

Supplementary antibody results

**eTable 1**: Characteristics of thymomatous MG patients with spontaneous muscle overactivity tested for neuronal autoantibodies

**eFigure 1:** staining patterns for LGI1, CASPR2 and AMPAR antibodies on rat brain tissue.

**Supplementary methods**

**Live cell-based assay for LGI1, Caspr2, UNC5A and GlyR**

8 mm round glass coverslips were placed at the bottom of a 6 well plate (8 coverslips/well), treated with poly-lysine (Sigma) and stored for later use. HEK293T cells were seeded in the 6 well plates at the density of 35x10^4^/well and grown using Dulbecco’s Modified Eagle Medium (DMEM) with fetal bovine serum and antibiotics/antimicotics. After 24 hours (CO_2_ incubator), when a confluency of 80% was reached, cells were transfected with the appropriate cDNA (3 ug/well) using lipofectamine 2000 (Invitrogen, Carlsbad, California, USA) according to manufacturer’s instructions. After 24 hours, cells were collected and used for staining. cDNA constructs for Caspr2, LGI1 and Gly receptor were kindly donated by Dr. Paddy Waters and Prof. Angela Vincent (Oxford University, UK). cDNA construct for UNC5A was purchased from OriGene (Rockville, Maryland, USA)

Live cells were incubated with patients’ sera at the optimal dilution, depending on the transfected plasmid (1:40 dilution for LGI1, GlyR and UNC5A, 1:100 dilution for Caspr2) in DMEM-HEPES-1% bovine serum albumin (BSA) for 45 minutes at room temperature.

Cells were then washed 3 times with DMEM-HEPES, fixed for 10 minutes with 4% cold paraformaldehyde (PFA), washed again 3 times with DMEM-HEPES, and incubated with fluorescent conjugated anti-human IgG Fc (Jackson scientific, West grove, Pennsylvania, USA) diluted 1:750 in DMEM-HEPES-1%BSA for 45 minutes.

Finally, cells were washed 2 times with DMEM-HEPES and 2 times with deionized H_2_O, and then mounted using a fluorescent mounting medium containing DAPI (Dako, Santa Clara, California, USA). Results were assessed using a fluorescence microscope.

**Fixed cell based assay for DCC antibodies**

HEK293T cells were prepared as described above for the live CBA, with the following differences: cells were transfected using a reduced amount of DCC cDNA (1.5 ug/well; kindly provided by Prof. Masaki Fukata). After 10 hours from the transfection (longer transfection times led to increased background staining and non-specific binding of the secondary antibody on transfected cells), cells were removed from the incubator, washed 3 times with PBS, fixed for 10 minutes with 4% cold PFA, and washed again 3 times with Phosphate Buffer Saline (PBS). Cells were then permeabilised for 5 minutes with 0.2% PBS-triton, and then blocked for 1 hour with PBS-5%BSA at room temperature. Afterwards, blocking solution was sucked completely from the wells, and slides were placed at -80°C until later use.

To perform the staining, slides were removed from the -80°C and incubated for 45 minutes with patient serum diluted 1:100 in PBS-5%BSA and a commercial rabbit anti-human antibody against DCC (Proteintech, Manchester, UK) diluted 1:500. Cells were then washed 3 times in PBS and incubated at room temperature with fluorescent conjugated anti-human IgG Fc (Jackson scientific, West grove, Pennsylvania, USA) diluted 1:2000 and Alexafluor 488 goat anti-rabbit IgG (Invitrogen) diluted 1:1000 in PBS-5%BSA for 45 minutes. Finally, cells were washed 3 times with PBS, 3 times with deionized H_2_O and then mounted using a fluorescent mounting medium containing DAPI. Results were assessed using a fluorescence microscope.

**Supplementary antibody results**

The 4 samples positive for LGI1 and/or Caspr2 antibodies were confirmed on immunohistochemistry, which showed a neuropilar pattern consistent with that of each antibody. Only 1 additional patient showed a hippocampal neuropilar staining, that was characterized as AMPAR antibodies in Barcelona Neuroimmunology Laboratory (Prof. Francesc Graus). The patient with isolated DCC antibodies showed no specific staining on immunohistochemistry.

eTable 1: Characteristics of thymomatous MG patients with spontaneous muscle overactivity tested for neuronal autoantibodies

| Patients, n (%) | 23 (100) |
| --- | --- |
| Months of follow-up (from NMT onset), median (IQR) | 45 (21-56) |
| Months of follow-up (from thymectomy), median (IQR) | 86 (62-121) |
| Males, n (%) | 8 (34.8) |
| Age at MG onset, median (IQR) | 49 (38-60.5) |
| MGFA ≥ III before thymectomy, n (%) | 7 (30.4) |
| Osserman ≥ IIb before thymectomy, n (%) | 15(65.2) |
| Latency from MG to NMT, months, median (IQR) | 45 (12-81) |
| Age at NMT presentation, mean (SD) | 46 (36-57) |
| Symptoms |  |
| *cramps,* n (%) | 20 (87) |
| *myokymias,* n (%) | 17(73.9) |
| *fasciculations,* n (%) | 10(43.5) |
| *muscle twitching* n (%) | 20(87.0) |
| Symptoms distribution |  |
| *cranial* n (%) | 13 (56.5) |
| *trunk* n (%) | 1 (4.4) |
| *upper limbs* n (%) | 11 (47.8) |
| *lower limbs* n (%) | 17 (73.9) |
| Additional Symptoms |  |
| *tremors* n (%) | 15 (62.5) |
| *muscular rigidity* n (%) | 10 (43.5) |
| *paresthesia* n (%) | 7 (30.4) |
| *pain* n (%) | 8 (34.8) |
| *weight loss* n (%) | 4 (17.4) |
| CNS involvement, n (%) | 8 (34.8) |
| *confusion/memory impairment,* n (%) | 3 (13.0) |
| *sleep disorders,* n (%) | 5 (21.7) |
| *seizures,* n (%) | 2 (8.7) |
| Dysautonomia, n (%) | 10 (43.5) |
| *hyperhidrosis,* n (%) | 9 (39.1) |
| *arrhythmias,* n (%) | 4 (17.4) |
| *gastrointestinal dysmotility,* n (%) | 3 (13.0) |
| Thymoma WHO (≥ 2B), n (%) | 12 (52.2) |
| Thymoma recurrence, n (%) | 8 (34.8) |
| NMT response to therapy, n (%) | 12/14 (85.7) |
| MG Outcome n (%) |  |
| *improved* | 5 (21.7) |
| *minimal manifestations* | 8 (34.8) |
| *pharmacological remission* | 7 (30.4) |
| *complete stable remission* | 2 (8.7) |
| *death* | 1 (4.3) |

Ab, antibody; NMT, neuromyotonia; IQR, interquartile range; MG, myasthenia gravis; MGFA, MG Foundation of America classification; WHO, World Health Organization score; SD, standard deviation.

**e-Figure 1: Staining patterns of LGI1, CASPR2, and LGI1 plus Caspr2 antibodies on rat brain tissue**


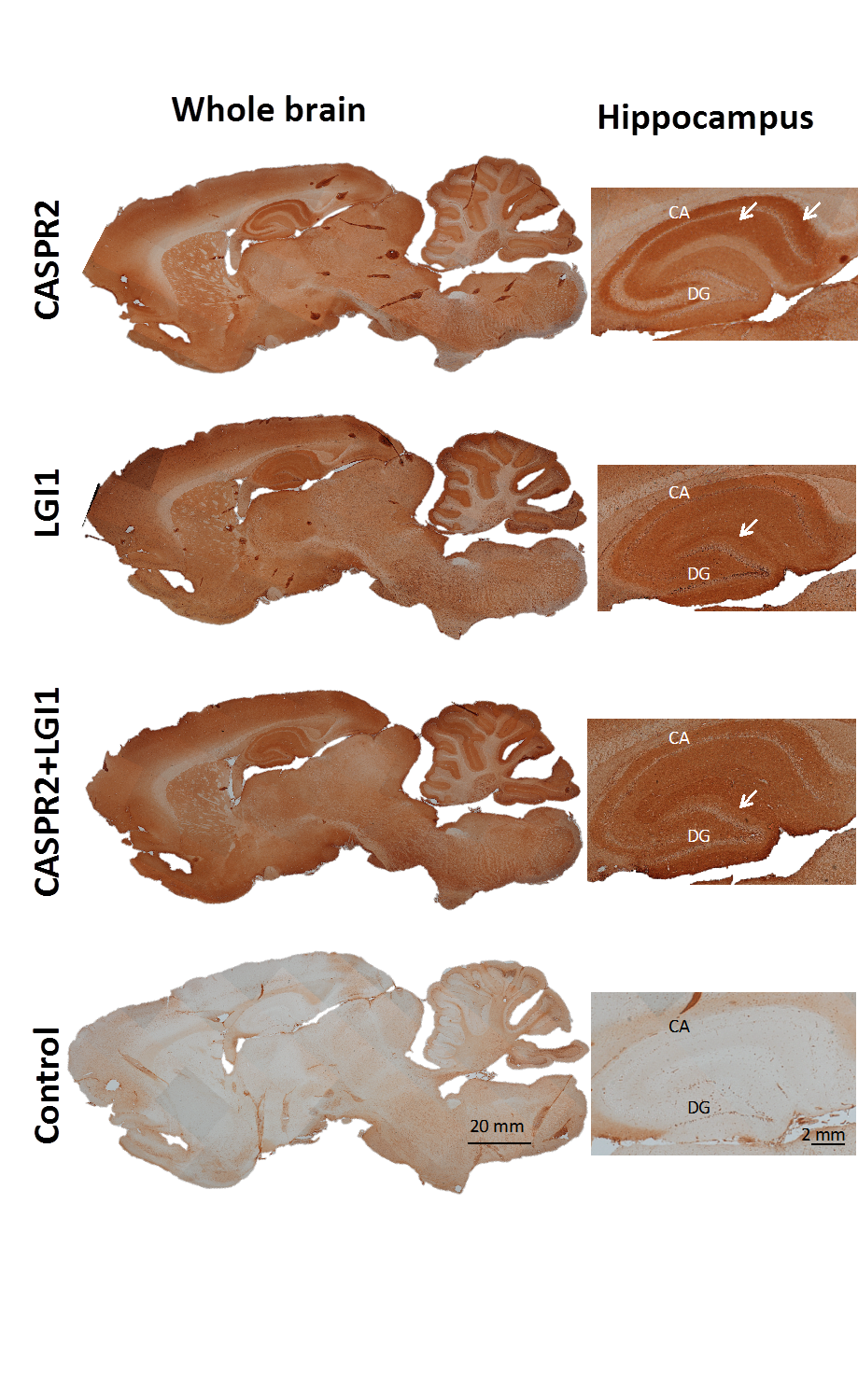


Examples of the staining patterns provided by sera (1:200 dilution) of thymomatous MG patients with NMT positive for Caspr2, LGI1 or both antibodies. Caspr2 provides a hippocampal staining more intensely localised in the Cornus Ammonis (CA) neuropilum (white arrows) compared to the Dentate Gyrus (DG). LGI1 provides a typical staining in the DG that involves more intensely the outer third of the molecular layer (white arrow). When Caspr2 and LGI1 antibodies coexist, the LGI1 staining tends to prevail, making the identification of both antibodies just from the staining pattern very difficult (white arrow). No neuropilar staining is present with a control serum.
